# Endoneurial immune interplay in peripheral nerve repair: insights and implications for future therapeutic interventions

**DOI:** 10.3389/fnins.2025.1602112

**Published:** 2025-05-09

**Authors:** Alina Sprenger-Svačina, Martin K. R. Svačina, Husniye G. Otlu, Tong Gao, Kazim A. Sheikh, Gang Zhang

**Affiliations:** ^1^Neuromuscular Research Laboratory, Department of Neurology, McGovern Medical School, The University of Texas Health Science Center at Houston, Houston, TX, United States; ^2^Department of Neurology, Faculty of Medicine, University Hospital of Cologne, Cologne, Germany; ^3^Philipps University Marburg and Department of Neurology, University Hospital Gießen and Marburg, Marburg, Germany; ^4^Vocational Health Sciences, Laboratory Techniques Program, Malatya Turgut Ozal University, Malatya, Türkiye

**Keywords:** macrophage, macrophage polarization, schwann cell, T cell, endoneurial inflammation, peripheral nerve injury, peripheral nerve repair, immunomodulation

## Abstract

The mechanisms underlying axonal injury and repair in peripheral nerves, whether due to traumatic damage or autoimmune neuropathies, are complex and not yet fully understood. Recent research indicates that an orchestrated interplay between damaged neurons, Schwann cells, and especially endoneurial immune cells such as macrophages or T cells is crucial to achieve satisfactory nerve recovery. Following axonal injury, degenerating axons and reactive Schwann cells release chemoattractants and cytokines that recruit immune cells into the endoneurium. Among them, macrophages play a pivotal role by clearing axonal and myelin debris and subsequently creating a pro-regenerative microenvironment that supports axonal outgrowth. There is evidence that the timely switch of a pro-inflammatory M1 toward a pro-regenerative M2 macrophage polarization state is crucial for satisfactory nerve recovery, and supportive cellular and humoral factors that influence the endoneurial microenvironment, such as T cells and their cytokines, can substantially impact this fragile recovery process. The latter explains the limited nerve recovery in immune neuropathies, where a pathologic pro-inflammatory shift within the endoneurial immune cell signature hampers axonal outgrowth. This review aims to provide insights into cellular and humoral determinants of the endoneurial microenvironment during nerve damage and repair, which are assumed to hold substantial potential for future therapeutic interventions, especially since current strategies to enhance peripheral nerve recovery are limited to either surgical interventions in traumatic neuropathies or immunomodulatory drugs in immune neuropathies that often fail to achieve satisfactory functional results.

## Introduction

Peripheral nerve injuries, whether resulting from traumatic events or autoimmune pathologies, pose a significant clinical challenge due to the complex nature of axonal damage and the limited regenerative capacity of the peripheral nervous system. The extent and site of axonal injury are critical determinants of the prognosis for nerve repair and functional recovery. While peripheral nerves possess an intrinsic ability to regenerate, the repair process is often inefficient and incomplete, leading to chronic functional deficits and long-term disability (Grinsell and Keating, 2014). The mechanisms underlying nerve repair involve a complex interplay of cellular and molecular processes, yet many aspects remain poorly understood. Advancing our knowledge of these processes could open new avenues for therapeutic innovation aimed at enhancing axonal regeneration and functional recovery.

Previous studies highlight the critical role of the endoneurial microenvironment in mediating nerve repair (Forbes and Rosenthal, 2014). The response to peripheral nerve injury (PNI) involves a highly coordinated interaction among damaged neurons, Schwann cells, resident immune cells, and infiltrating immune cell populations (Scheib and Hoke, 2013; Forbes and Rosenthal, 2014). These cells work collectively to clear axonal and myelin debris, resolve inflammation, and establish a regenerative environment that supports axonal regrowth. Among these key cellular mediators, macrophages play a pivotal role due to their remarkable plasticity and ability to transition between distinct functional states (Kigerl et al., 2009; Chen et al., 2015; Wynn and Vannella, 2016). This polarization is not binary but occurs along a spectrum, broadly classified as: pro-inflammatory (M1-like) macrophages and pro-regenerative (M2-like) macrophages. This dynamic and tightly regulated polarization ensures a sequential immune response, where initial inflammation clears debris and prevents infection, followed by a pro-regenerative phase that fosters axonal outgrowth and remyelination. The successful transition from inflammation to regeneration is heavily influenced by Schwann cells and damaged axons, which secrete key signaling molecules that guide macrophage activity within the endoneurium (Shamash et al., 2002; Martini et al., 2008; Rotshenker, 2011).

Beyond macrophages, other immune cells, including T cells and neutrophils, contribute to nerve repair process by either directly interacting with macrophages or shaping the broader inflammatory milieu (Lindborg et al., 2017; Hu et al., 2018; Bombeiro et al., 2020a; Tang et al., 2022). Notably, T cells secrete cytokines that can either support or hinder repair, depending on the prevailing immune balance. This delicate equilibrium underscores the importance of a timely resolution of inflammation, as a sustained pro-inflammatory state can disrupt repair processes and exacerbate tissue damage. This phenomenon is particularly evident in autoimmune neuropathies, where an aberrant immune response perpetuates inflammation, impairs extracellular matrix (ECM) remodeling, and inhibits axonal regeneration (Kigerl et al., 2009; Wynn and Vannella, 2016).

Current therapeutic strategies for peripheral nerve injuries are limited in scope and efficacy (Lopes et al., 2022). Surgical repair remains the primary option for traumatic nerve injuries, while immunomodulatory therapies are the mainstay for autoimmune neuropathies. However, both approaches often yield suboptimal functional recovery, highlighting an urgent need for alternative strategies that target the underlying biological processes of nerve repair. Emerging evidence suggests that modulating the immune response, particularly the activity of endoneurial macrophages, holds significant promise for enhancing nerve repair and functional outcomes (Dubovy et al., 2013; Wynn and Vannella, 2016).

This review explores the cellular and molecular factors shaping the endoneurial microenvironment during nerve injury and repair, with a focus on the role of macrophages and their interactions with other immune cells and Schwann cells. By understanding these mechanisms, we aim to identify novel therapeutic opportunities that can overcome current limitations. Key areas of focus include fostering a pro-regenerative immune environment through targeted macrophage polarization, selectively modulating immune cell subsets to enhance axonal repair, and integrating immunomodulatory strategies with advanced surgical and biomaterial approaches to improve clinical outcomes. Leveraging these insights may lead to the development of novel interventions that more effectively promote axonal regrowth and functional recovery in patients with peripheral nerve injuries.

### Mechanisms of peripheral nerve injury

Peripheral nerve injury can manifest as either an acute or chronic condition, with a wide spectrum of clinical subtypes and diverse underlying causes. The most common causes of PNI characterized by predominant axonal damage include metabolic disorders (e.g., diabetes mellitus), toxic exposures (e.g., chronic alcohol abuse, chemotherapeutic agents), and trauma (e.g., nerve crush or transection) (Sommer et al., 2018). Additionally, autoimmune diseases (e.g., Guillain-Barré syndrome) and hereditary neuropathies (e.g., Charcot-Marie-Tooth disease type 2) can also result in axonal injury in peripheral nerves (Collins et al., 2010; Vrancken and Said, 2013; Kieseier et al., 2018; Naum and Gwathmey, 2023; Alberti et al., 2024). While PNI encompasses a broad range of clinical subtypes, this review will specifically focus on autoimmune neuropathies and traumatic peripheral nerve injury.

Axonal damage in PNI can result from various mechanisms, including: (1). Microcirculatory disruption: impaired blood supply in conditions like diabetes mellitus and vasculitis leads to ischemic damage and axonal degeneration (Collins and Periquet, 2008; Horton and Barrett, 2021); (2). Dysfunctional axonal transport or energy metabolism: chemotherapy, chronic alcoholism, and hereditary neuropathies can disrupt mitochondrial function and cytoskeletal transport, impairing axonal integrity (Maiya and Messing, 2014; Alberti et al., 2024); (3). Direct axonal damage: traumatic injuries (axonotmesis or neurotmesis) cause mechanical disruption, while reactive oxygen species (ROS), generated in chemotherapy-induced, diabetic, alcoholic, inflammatory, and vasculitic neuropathies (Yu et al., 2024; Ou et al., 2025), exacerbate axonal injury.

Following axonal damage, Wallerian degeneration is initiated to clear damaged axons and prepare the microenvironment for regeneration (Icso and Thompson, 2022). Wallerian degeneration is a tightly regulated process involving axonal fragmentation, myelin degradation, macrophage infiltration, and Schwann cell activation. Disruptions in Wallerian degeneration, such as prolonged inflammation or impaired macrophage-mediated clearance, can lead to chronic neuropathic conditions and failed nerve regeneration.

In contrast to axonal neuropathies, inflammatory demyelinating polyneuropathies, such as chronic inflammatory demyelinating polyneuropathy (CIDP) and the acute inflammatory demyelinating polyneuropathy (AIDP) variant of Guillain-Barré syndrome (GBS), are characterized by primary myelin damage, which may lead to secondary axonal injury over time (Bunschoten et al., 2019; Shahrizaila et al., 2021). In these conditions, an autoimmune response against myelin antigens (Khalili-Shirazi et al., 1993; Louvet et al., 2009) triggers macrophage-mediated destruction of the myelin sheath, which is carried out via the release of ROS, pro-inflammatory cytokines, and myelin phagocytosis (Zigmond and Echevarria, 2019). Secondary to myelin breakdown, axonal degeneration may take place, with variable severity depending on the duration and extent of inflammatory demyelination (Alcantara et al., 2021; Quint et al., 2024). The autoimmune response against peripheral nerve myelin is orchestrated via autoreactive T cells, macrophages, and autoantibodies secreted by B cells (Shahrizaila et al., 2021; Svacina et al., 2024b), but in most cases, the exact target antigens remain currently unknown. Persistent immune-mediated demyelination leads to progressive nerve dysfunction, contributing to chronic disability in patients with CIDP and other autoimmune neuropathies.

In AIDP, evidence suggests that secondary axonal damage is associated with disruptions in nodal architecture and cytoskeletal organization. Studies conducted in the Experimental Autoimmune Neuritis (EAN) model have demonstrated that voltage-gated sodium and potassium channels, along with AnkyrinG, become disorganized before the loss of Neurofascin-186 (NF-186) and Gliomedin from the node of Ranvier (Lonigro and Devaux, 2009). Autoantibodies targeting NF-186 and Gliomedin in these models suggest a potential autoimmune mechanism underlying axonal injury. Additionally, increased neurofilament density has been observed in peripheral nerve axons of AIDP patients, indicating cytoskeletal abnormalities (Carpenter, 1972). Similarly, CIDP models reveal disturbed nodal architecture and axonal cytoskeletal organization as contributors to secondary axon degeneration. Biopsy samples from CIDP patients show disorganization of voltage-gated sodium channels, contactin-associated protein (CASPR), paranodin, and NF proteins (Cifuentes-Diaz et al., 2011; Doppler et al., 2015; Doppler et al., 2016). In rats treated with contactin 1 (CNTN1) IgG4 antibodies, CASPR, CNTN1, and NF-155 organization was disrupted, reinforcing the role of autoantibodies in axonal pathology (Manso et al., 2016). Furthermore, CIDP patient biopsies show increased neurofilament and microtubule densities, mitochondrial accumulation, and altered neurofilament phosphorylation, indicating impairments in axonal transport and cytoskeletal integrity (Prineas and McLeod, 1976; Vital et al., 2000). These findings collectively suggest that nodal architecture disruption, cytoskeletal disorganization, and axonal transport deficits contribute to axonal degeneration in inflammatory demyelinating neuropathies.

### Macrophages as key regulators of nerve repair

Macrophages are central to nerve repair, mediating debris clearance, Schwann cell activation, and axonal regeneration (Wynn and Vannella, 2016). They exist as tissue-resident macrophages (TRMs) and blood-derived macrophages (BDMs) and functionally polarize into pro-inflammatory (M1-like) and anti-inflammatory/pro-repair (M2-like) states. Macrophage polarization, driven by environmental cues, is critical for immune response, tissue repair, and disease progression. M1 macrophages, induced by Toll-like receptor (TLR) ligands and interferon-gamma (IFN-γ), initiate inflammation and pathogen clearance (Wynn and Vannella, 2016). M2 macrophages support regeneration and exist in subtypes (Gordon, 2003; Chen et al., 2015): M2a (IL-4/IL-13-induced) enhances cell proliferation and apoptotic cell clearance; M2b (immune complex-induced) promotes angiogenesis and tissue stabilization; M2c (IL-10/TGF-β-induced) resolves inflammation and aids repair; and M2d (A2AR agonist-induced) facilitates angiogenesis and wound healing. Macrophages are highly plastic and can switch between M1 and M2 states in response to environmental changes (Biswas and Mantovani, 2010; Locati et al., 2020). This dynamic regulation maintains immune balance and supports efficient tissue recovery.

Upon peripheral nerve damage, damaged axons and Schwann cells release a variety of factors that initiate an endoneurial immune response (Martini et al., 2008; Rotshenker, 2011). Animal studies in the sciatic nerve crush model, which is used to examine peripheral nerve repair by completely disrupting axonal integrity via crush injury, but preserving endoneurial fibrous structures like the perineurium as lead structures of regeneration within the sciatic nerve (Perry et al., 1987; Bendszus and Stoll, 2003; Svacina et al., 2024a), suggest that the post-injury immune response incorporates a crucial two-step process for a successful nerve repair: (I) Within the first 7–10 days after injury, the distal axonal segment undergoes Wallerian degeneration, leading to the breakdown of axons and myelin. A pro-inflammatory immune response arises within the endoneurium, recruiting macrophages and neutrophils for axonal and myelin debris clearance. Early neovascularization begins, supplying nutrients and oxygen to the regenerating nerve; (II) Thereafter, an immunologic shift toward the resolution of inflammatory cascades in favor of a pro-regenerative microenvironment, characterized by the production of angiogenic and neurotrophic factors, enables successful peripheral nerve repair (Nadeau et al., 2011; Ydens et al., 2012). This transition is critical, as prolonged inflammation hinders axonal regrowth, contributing to poor recovery outcomes in conditions like immune neuropathies and aging (Scheib and Hoke, 2016; Buttner et al., 2018; Olsen et al., 2024; Sprenger-Svacina et al., 2024b; Sprenger-Svacina et al., 2024c; Svacina et al., 2024a).

The transition from M1 to M2 macrophages is governed by a complex interplay of microenvironmental cues. In autoimmune polyneuropathies, the timely conversion from M1 to M2 fails due to several interrelated factors. One key reason is the persistence of pro-inflammatory cytokine signaling, which sustains M1 polarization and prevents the resolution of inflammation. Chronic activation driven by autoantigens may also contribute, as continuous exposure to myelin antigens keeps macrophages in a pro-inflammatory state, restricting their plasticity. Additionally, dysfunctional regulatory mechanisms, particularly Treg dysfunction, lead to impaired secretion of IL-10 and TGF-β, further sustaining inflammation and preventing the shift to an anti-inflammatory phenotype. Understanding these disruptions in M1-to-M2 conversion is essential for developing targeted interventions to mitigate persistent inflammation and promote nerve repair.

Injury-induced chemokines such as C-C motif chemokine ligand 2 (CCL2) and fractalkine [also known as C-X3-C motif ligand 1 (CX3CL1)], attract monocytes to the endoneurium, where they differentiate into BDMs upon adhesion to vascular endothelium (via CX3CL1/CX3CR1 interaction) (Tanaka et al., 2020) and subsequent transmigration to the endoneurium (via CCL2/CCR2 axis) (Liu et al., 2019). In the early post-injury phase, BDMs polarize toward an M1 phenotype, driven by damage-associated molecular patterns (i.e., proteins derived from endoneurial debris) binding to TLRs (Liu et al., 2019). Furthermore, the attraction of neutrophils to the distal nerve stump via C-X-C motif chemokine ligands 1 and 2 (CXCL1 and CXCL2) chemokine release, which peaks as early as 3 days post-injury, boosts the clearance of endoneurial debris (Lindborg et al., 2017).

A disruption of endoneurial blood vessels, alongside the release of pro-inflammatory factors [e.g., CCL2, interleukin-1alpha (IL-1α) and IL-1β, tumor necrosis factor-alpha (TNF-α)] by damaged axons and Schwann cells, and boosted by the recruited leukocyte populations, induces hypoxic conditions within the damaged endoneurium (Lim et al., 2015). Resident and infiltrating macrophages have the capability to sense this hypoxic environment, and react via the release of hypoxia-inducible factor-1 alpha (HIF-1α), which is synthetized by pro-inflammatory M1 macrophages (Hollander et al., 2001; Liu et al., 2019). Although a recent study on human BDMs from gastric cancer patients suggests that hypoxia favors the anti-inflammatory M2 phenotype (Poirah et al., 2024), in peripheral nerve inflammation, hypoxia more likely drives an initial pro-inflammatory shift toward M1 polarization. This is supported by the observation that only M1 macrophages have been reported to produce HIF-1α under hypoxic conditions in the endoneurium (Liu et al., 2019). The induction of HIF-1α in macrophages further promotes vascular endothelial growth factor (VEGF) expression, facilitating neovascularization to support axonal regeneration (Pugh and Ratcliffe, 2003).

Macrophages play a vital role in ECM remodeling and fibrosis prevention (Wynn and Vannella, 2016), both essential for axonal regrowth in peripheral nerve repair. Fibrosis, the excessive accumulation of collagen and other ECM proteins, creates a physical barrier that hinders nerve regeneration. Macrophages maintain ECM homeostasis by secreting proteolytic enzymes, cytokines, and growth factors that influence ECM composition and fibroblast activity (Wynn and Vannella, 2016). A key mechanism involves the secretion of matrix metalloproteinases (MMPs), which degrade excessive ECM components and clear fibrotic tissue that may obstruct axonal regeneration. Simultaneously, macrophages secrete tissue inhibitors of metalloproteinases (TIMPs) to tightly control MMP activity, preventing excessive ECM degradation and maintaining tissue integrity (Khokha et al., 2013). Macrophages also influence fibroblast behavior through cytokine signaling (Buechler et al., 2021). TGF-β, when secreted in a controlled manner, regulates fibroblast activity, ensuring balanced ECM deposition without excessive scarring. Myofibroblasts, which contribute significantly to fibrosis by producing large amounts of ECM and alpha-smooth muscle actin, are also targets of macrophage regulation. By influencing myofibroblast apoptosis and ECM turnover, macrophages help prevent excessive scarring and support tissue remodeling (Wynn and Barron, 2010). When macrophage-mediated ECM remodeling is disrupted, excessive fibrosis can result in a dense collagenous scar that obstructs axonal regrowth.

**FIGURE 1:**
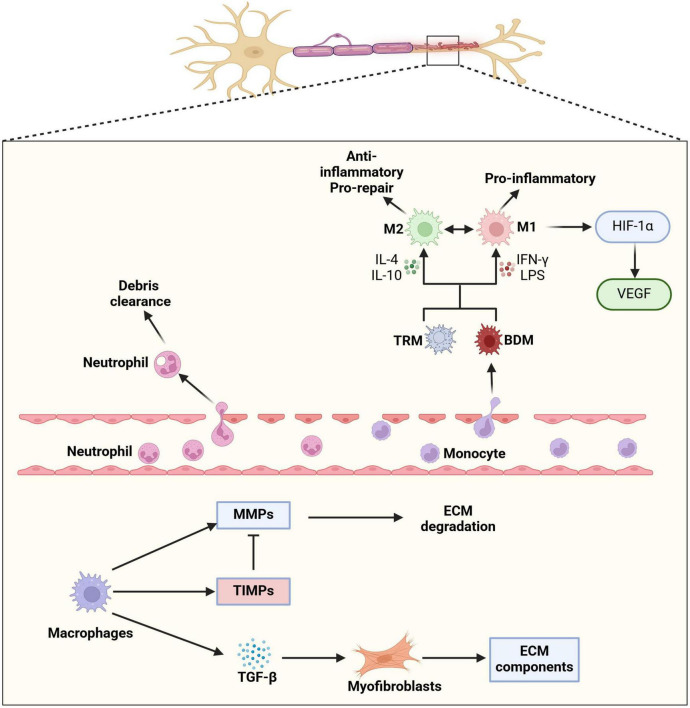
Macrophage involvement following peripheral nerve injury. Following peripheral nerve injury, the local microenvironment undergoes significant changes that drive macrophage recruitment, differentiation, and functional polarization. Injury-induced chemokines, such as CCL2 and CX3CL1, mediate the recruitment of neutrophil and circulating monocytes into the endoneurium. Once within the nerve tissue, these monocytes differentiate into blood-derived macrophages (BDMs), which work alongside pre-existing tissue-resident macrophages (TRMs) to orchestrate the immune response. Macrophage polarization is a highly dynamic and context-dependent process, influenced by cytokines, cellular interactions, and metabolic cues. Pro-inflammatory (M1) macrophages, induced by IFN-γ, contribute to early immune responses by releasing inflammatory cytokines and reactive oxygen species (ROS) that help clear cellular debris. In contrast, anti-inflammatory (M2) macrophages, stimulated by IL-4 and IL-10, support tissue repair by promoting extracellular matrix (ECM) remodeling, angiogenesis, and axonal regrowth. The ability of macrophages to switch between M1 and M2 phenotypes in response to environmental signals is crucial for maintaining immune homeostasis and ensuring effective nerve regeneration. This plasticity allows macrophages to fine-tune their functions throughout different phases of nerve repair. In particular, M1 macrophages initiate the inflammatory response and phagocytosis of myelin debris, while M2 macrophages facilitate fibrosis resolution and ECM remodeling through secretion of matrix metalloproteinases (MMPs) and fibrotic regulators such as TGF-β. Through the regulation of ECM components and modulation of myofibroblast activity, macrophages help control the deposition and breakdown of fibrotic tissue, thereby preventing excessive scarring that could hinder nerve regeneration. The coordinated actions of different macrophage subsets create an optimal microenvironment for axonal regrowth and functional recovery.

Macrophages orchestrate a finely tuned immune response essential for peripheral nerve regeneration, balancing inflammation, ECM remodeling, and fibrosis prevention (Figure 1). A timely switch from M1 to M2 macrophages is crucial for successful nerve repair (Mokarram et al., 2012; Liu et al., 2019), making macrophage-targeted interventions a potential therapeutic avenue for improving outcomes in traumatic and autoimmune neuropathies.

### The role of Schwann cells and other immune cells in peripheral nerve repair

Schwann cells play a pivotal role in peripheral nerve repair by undergoing a phenotypic transformation that supports regeneration. Following nerve injury, they transition from a mature myelinating or non-myelinating state to a proliferative, precursor-like “repair” Schwann cell phenotype, orchestrating several key regenerative processes. One of their primary functions is debris clearance. Repair Schwann cells facilitate the removal of myelin fragments via autophagy and recruit macrophages to assist in myelin debris clearance through macrophage-mediated phagocytosis (Hirata and Kawabuchi, 2002; Gomez-Sanchez et al., 2015; Brosius Lutz et al., 2017). This process is essential, as lingering myelin debris can impede axonal regrowth.

Beyond debris removal, Schwann cells provide both structural and biochemical support for regenerating axons. They align with newly formed blood vessels, creating a scaffold that directs axonal extension. This is achieved through the production of extracellular matrix (ECM) components such as collagen IV, elastin, and fibronectin, along with myelin-associated glycoprotein, peripheral myelin proteins, and adhesion molecules like neuronal cell adhesion molecule. These elements establish a stable basal lamina that anchors and supports regenerating axons. Additionally, Schwann cells release neurotrophic factors, including nerve growth factor and brain-derived neurotrophic factor, which guide axonal outgrowth at an estimated rate of 1 mm/day in humans (Jiang et al., 2024).

Schwann cells also contribute to immune modulation during peripheral nerve injury. Following nerve injury, they upregulate pro-inflammatory cytokines such as TNF-α, IL-1β, and IL-6, and secrete chemokines like CCL2, which activate local macrophages and help recruit immune cells, including macrophages and neutrophils (Shamash et al., 2002; Tofaris et al., 2002). On the other hand, Schwann cells also produce anti-inflammatory IL-10 to regulate the immune responses and prevent excessive inflammation (Zhang et al., 2011). In addition to cytokine signaling, Schwann cells express pattern recognition receptors (PRRs), such as TLRs, that recognize damage-associated molecular patterns (DAMPs) and trigger innate immune responses (Goethals et al., 2010). While Schwann cells primarily modulate the innate immune response, they also interact with the adaptive immune system by presenting antigens via MHC-II to T cells (Meyer zu Horste et al., 2010). In autoimmune neuropathies, this antigen presentation can contribute to aberrant immune activation and sustained inflammation. By coordinating inflammation, debris clearance, scaffold formation, and neurotrophic support, Schwann cells act as both architects and mediators of peripheral nerve repair, ensuring successful nerve regeneration.

In addition to macrophages and Schwann cells, other immune cells, such as neutrophils and T cells, play crucial roles in the peripheral nerve repair process (Lindborg et al., 2017; Bombeiro et al., 2020a; Tang et al., 2022). These cells contribute to the repair process through direct interactions with macrophages or by modulating the broader inflammatory microenvironment, thereby influencing the balance between inflammation and regeneration.

Neutrophils are the first immune cells to infiltrate the injury site, playing a vital role in early myelin debris clearance and inflammatory signaling (Lindborg et al., 2017). While beneficial in the acute phase, their prolonged presence can be detrimental. Neutrophils not only recruit inflammatory monocytes but also amplify their pro-inflammatory effects by promoting M1 macrophage polarization, enhancing cytokine release, and increasing ROS production (Soehnlein et al., 2009a; Soehnlein et al., 2009b). This neutrophil-monocyte axis sustains and exacerbates inflammation in various pathological conditions. Effective clearance of neutrophils by macrophages is essential for shifting toward a regenerative immune environment (Huynh et al., 2002; Filardy et al., 2010).

T cells are increasingly recognized as key mediators of peripheral nerve injury and repair, with their effects largely dictated by their subtype and activation state (Bombeiro et al., 2020b; Tang et al., 2022). Pro-inflammatory T cells (Th1 and Th17 subsets) secrete IFN-γ and IL-17, which enhance macrophage M1 polarization and sustain the inflammatory response. These cells also recruit additional immune cells, potentially exacerbating tissue damage if inflammation becomes chronic, as seen in autoimmune neuropathies where they contribute to myelin breakdown and axonal degeneration (Baxi et al., 2015). Conversely, pro-regenerative Th2 cells secrete IL-4 and IL-13, promoting M2 macrophage polarization and tissue repair (Hendrix and Nitsch, 2007). Additionally, regulatory T cells (Tregs) produce IL-10 and IL-4, which suppress excessive inflammation, modulate macrophage polarization, support ECM remodeling, and promote wound healing (Roszer, 2015; Witherel et al., 2021; Chen et al., 2022). These pro-regenerative T cells facilitate Schwann cell function, promoting remyelination and axonal regeneration. The delicate equilibrium between these pro-inflammatory and pro-regenerative T cell subsets determines whether the immune response resolves efficiently or leads to chronic dysfunction. A persistent Th1/Th17-driven inflammatory state, as seen in inflammatory neuropathies, prevents the timely resolution of inflammation, impairs ECM remodeling, and hinders axonal regeneration, contributing to chronic nerve degeneration.

The interplay between Schwann cells, T cells, neutrophils, and macrophages is essential for orchestrating an effective repair response following peripheral nerve injury. While pro-inflammatory immune activity is necessary for pathogen defense and debris clearance, its persistence can exacerbate tissue damage and impair regeneration. Conversely, pro-regenerative immune responses promote remyelination, ECM remodeling, and axonal regrowth. A precise immune balance is therefore critical for successful nerve repair, highlighting the potential of immune-targeted therapies in treating peripheral nerve injuries and neuropathies.

### Immunological mechanisms and impediments to nerve repair

As discussed above, peripheral nerve repair is a complex process influenced by various immunological mechanisms. While inflammation is essential for successful regeneration, dysregulated or excessive immune responses can significantly hinder recovery. A tightly controlled balance of pro- and anti-inflammatory immune activity within the first month after injury is critical for orchestrating the subsequent regenerative processes. Macrophages, the predominant leukocyte population within the endoneurium, play a central role in regulating these delicate pro- and anti-inflammatory processes and are key modulators of the endoneurial inflammatory milieu (Liu et al., 2019).

Several factors can disrupt peripheral nerve regeneration. First, inadequate clearance of endoneurial debris during the initial inflammatory phase can lead to prolonged, inefficient hyperinflammation, hindering axonal outgrowth. This phenomenon, observed in preclinical models of PNI using aging animals, is referred to as “inflammaging” (Buttner et al., 2018; Svacina et al., 2024a). Conversely, insufficient macrophage recruitment to the injured nerve, as seen in CCL2/CCR2-deficient mice or immunocompromised individuals, also impairs repair (Mocchetti et al., 2014; Pan et al., 2020). This is because the initiation of the macrophage-driven inflammatory cascade, essential for clearing debris and preparing the path for axonal regrowth, is compromised.

Second, persistent endoneurial inflammation with inadequate resolution, characteristic of inflammatory neuropathies, negatively impacts nerve repair. In these conditions, a substantial influx of pro-inflammatory (M1) macrophages into the endoneurium, whether acute (as in GBS or corresponding animal models) (Leonhard et al., 2024) or chronic [as in CIDP or the spontaneous autoimmune peripheral polyneuropathy (SAPP) mouse model] (Ubogu et al., 2012; Bunschoten et al., 2019), contributes to axonal damage. This damage can occur through several mechanisms: autoantibodies targeting axonal structures (as observed in some axonal variants of GBS), direct attack on axonal components by M1 macrophages and T cells, or, more commonly, macrophage-mediated inflammatory demyelination leading to secondary axonal damage (as seen in demyelinating GBS and CIDP) (Leonhard et al., 2024; Sprenger-Svacina et al., 2024c). Studies in EAN, an animal model of inflammatory demyelinating neuropathies, have demonstrated that prolonged M1 macrophage responses hinder nerve recovery and worsen clinical outcomes (Du et al., 2020), while promoting M2 macrophage polarization reduces axonal damage and improves clinical status (Han et al., 2016a). Furthermore, in the SAPP animal model and CIDP patients, therapeutic modulation of pro-inflammatory Fc-gamma receptors on monocytes/macrophages has shown promise, ameliorating disease progression and reducing axonal loss, highlighting the role of dysregulated endoneurial inflammation in impaired nerve repair (Zhang et al., 2019; Svacina et al., 2024b). In these immune-mediated neuropathies, persistent M1 macrophage-driven hyperinflammation, which remains unresolved due to defective concurrent anti-inflammatory mechanisms, creates an unfavorable microenvironment that obstructs successful nerve repair.

Finally, disruption of the blood-nerve barrier (BNB) and ineffective Schwann cell responses also impede nerve regeneration. The BNB, formed by endoneurial microvascular endothelial cells, regulates immune-neural interactions and maintains peripheral nerve homeostasis. Unlike the blood-brain barrier, which is regulated by astrocytes, the BNB lacks an equivalent glial component, and emerging evidence suggests that pericytes play a critical role in regulating its barrier function (Shimizu et al., 2008; Shimizu et al., 2011). Peripheral nerve pericytes contribute to BNB integrity and may modulate immune cell infiltration following injury or in autoimmune conditions. Moreover, these cells express and secrete neurotrophic factors (Shimizu et al., 2011), which may support axonal regeneration and Schwann cell function. Injury or autoimmunity can compromise the BNB, leading to excessive immune cell infiltration and prolonged inflammation. In conditions like CIDP and GBS, BNB breakdown results in the infiltration of pathogenically relevant hematogenous leukocytes in peripheral nerves and facilitates autoantibody and complement-mediated nerve damage (Kieseier et al., 2004; van den Berg et al., 2014; Dalakas, 2015; Willison et al., 2016). Given their dual role in maintaining barrier function and promoting regeneration, pericytes represent a promising target for strategies aimed at restoring BNB integrity and enhancing peripheral nerve repair. Schwann cell dysfunction, whether due to chronic inflammation or age-related decline, results in poor remyelination and inadequate support for regenerating axons (Buttner et al., 2018; Balakrishnan et al., 2020). In some autoimmune neuropathies, Schwann cells themselves are targets of immune attack, further exacerbating the repair process (Kwa et al., 2003; Querol et al., 2017).

### Therapeutic opportunities to enhance peripheral nerve repair

**FIGURE 2:**
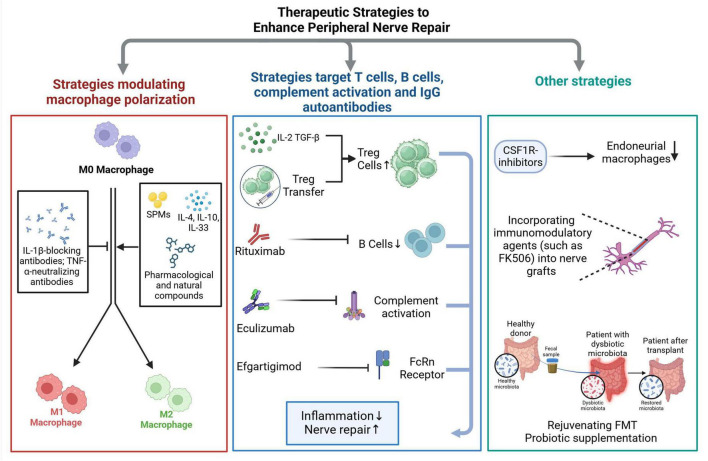
Summary of immunomodulatory strategies for peripheral nerve repair. Peripheral nerve injuries trigger complex immune responses that can either support or hinder nerve regeneration. Immunomodulatory therapeutic strategies aim to fine-tune these responses to promote functional recovery by enhancing axonal regeneration and minimizing secondary damage. The proposed strategies primarily focus on modulating key immune cell populations involved in nerve repair. One approach involves directing macrophage polarization to favor a pro-regenerative phenotype, as macrophages play a crucial role in clearing debris and providing trophic support to regenerating axons. Another set of strategies targets adaptive immune components, including T cells and B cells, as well as mechanisms involved in complement activation and the action of IgG autoantibodies, which have been implicated in inflammatory neuropathies. Beyond immune cell modulation, strategies that influence systemic factors, such as gut microbiota, are gaining attention. Emerging evidence suggests that interventions like fecal microbiota transplantation (FMT) or probiotic supplementation can reshape immune responses, potentially reducing inflammation and creating a more favorable environment for nerve regeneration. Another promising avenue involves blocking key pro-inflammatory signaling pathways, such as the colony-stimulating factor 1 (CSF1)/CSF1 receptor (CSF1R) axis, which is critical for macrophage survival, activation, and recruitment. Experimental models have shown that inhibiting this pathway can mitigate excessive inflammation and enhance regenerative outcomes.

Enhancing peripheral nerve repair is a major focus in regenerative medicine, particularly for traumatic peripheral nerve injury and autoimmune polyneuropathies. The extent of injury determines the therapeutic approach, ranging from conservative management for mild cases to surgical repair and advanced regenerative therapies for severe injuries (Lee and Wolfe, 2000; Campbell, 2008; Lopes et al., 2022). Current strategies for treating PNIs focus on restoring nerve continuity, enhancing axonal regeneration, and modulating the immune response to optimize functional recovery. Future immunomodulatory approaches seek to fine-tune immune activity to foster regeneration while minimizing excessive inflammation and fibrosis. These strategies target macrophage polarization, IgG autoantibodies, immune cell recruitment, and complement activation to create a pro-regenerative environment (Figure 2).

Regulating M2 macrophage polarization is a key strategy for promoting tissue repair and modulating immune responses, particularly in autoimmune polyneuropathies and peripheral nerve injuries. This process is primarily influenced by the JAK-STAT, TGF-β, and PPARγ signaling pathways, along with various metabolic mechanisms (Wynn and Vannella, 2016). Multiple pharmacological and biological approaches have been explored to enhance M2 polarization in experimental models (Table 1).

**TABLE 1:** The proposed therapeutic strategies that modulate macrophage polarization.

Strategy	Effect on macrophage polarization	References
IL-4 and IL-10 cytokines	Promote M2 macrophage polarization	Shen et al., 2022; Xin et al., 2022
Specialized Pro-resolving Mediators (SPMs)	Promote M2 macrophage polarization	Tang et al., 2013; Hong et al., 2014
Mesenchymal stem cells	Promote M2 macrophage polarization	Siniscalco et al., 2010
Metformin	Promotes M2 macrophage polarization	Zhou et al., 2023
siRNA against the transcription factor IRF-5	Inhibits M1 macrophage polarization	Goren et al., 2007; Courties et al., 2014
TNF-α-neutralizing antibodies	Inhibit M1 macrophage polarization	Goren et al., 2007; Courties et al., 2014
NLRP3 inflammasome inhibitors	Downregulate the M1 phenotype, facilitate an M2-like switch	Mirza et al., 2013; Mirza et al., 2014
IL-1β-blocking antibodies	Downregulate the M1 phenotype, facilitate an M2-like switch	Mirza et al., 2013; Mirza et al., 2014
PPAR-γ agonists	Promote M2 macrophage polarization	Mirza et al., 2015
DMF (Dimethyl fumarate)	Promotes M2 macrophage polarization	Han et al., 2016b
Compound A	Promotes M2 macrophage polarization	Zhang et al., 2009
Everolimus (RAD001)	Promotes M2 macrophage polarization	Han et al., 2016a
Bowman-Birk inhibitor concentrate (BBIC)	Promotes M2 macrophage polarization	Jin et al., 2016

Strategies to modulate macrophage phenotype have shown promise in regenerative medicine and could be applied to peripheral nerve repair. One approach involves inhibiting the inflammatory M1-like macrophage phenotype by blocking inflammatory cytokines such as IL-1β, IL-17, and TNF-α. Local administration of IL-1β-blocking antibodies or inhibitors of the NLRP3 inflammasome has been shown to downregulate the M1-like phenotype, facilitate an M2-like switch, and improve healing in diabetic mice (Mirza et al., 2013; Mirza et al., 2014). Similarly, systemic administration of TNF-α-neutralizing antibodies or nanoparticle delivery of siRNA against the transcription factor IRF-5, which is associated with M1-like activation, has been effective in reducing inflammation and promoting tissue repair in diabetic mice and myocardial infarction models (Goren et al., 2007; Courties et al., 2014).

Another strategy focuses on promoting the M2-like macrophage phenotype to enhance tissue repair. Administration of IL-4, either intraperitoneally or topically, has been shown to increase M2-like macrophage numbers, improve fibroblast activation, and accelerate healing in myocardial infarction and wound models, though its effects may extend beyond macrophages (Salmon-Ehr et al., 2000; Shiraishi et al., 2016). In spinal cord injury models, cytokines such as IL-4 and IL-10 promote M2 polarization, and nanoparticles or biomaterials can be used to deliver these cytokines directly to macrophages in injured nerves (Shen et al., 2022; Xin et al., 2022). IL-10 delivery via hydrogel scaffolds or lipid nanoparticles has shown promise in reducing fibrosis and improving nerve function (Shen et al., 2022). Additionally, IL-33 has been reported to enhance M2-like macrophage activity and accelerate wound closure (Yin et al., 2013). Lipid mediators, including specialized pro-resolving mediators (SPMs) such as resolvins, protectins, maresins, and lipoxin-A4, provide another means of modulating macrophage function. These molecules promote inflammation resolution by enhancing M2-like macrophage activity, facilitating wound healing in diabetic models (Tang et al., 2013; Hong et al., 2014), and reducing inflammation in corneal and tendon injuries (Kakazu et al., 2012; Dakin et al., 2015). Furthermore, activation of PPAR-γ, which is impaired in wound macrophages of diabetic mice and humans, has been shown to promote a M2-like phenotype. Topical application of PPAR-γ agonists restored macrophage function and improved healing in diabetic wounds (Mirza et al., 2015).

In addition to pharmacological interventions, macrophage-based cell therapy, involving transplantation of *ex vivo*-modified macrophages with a pro-regenerative phenotype, has been explored in spinal cord injury models and holds potential for peripheral nerve repair (Van Broeckhoven et al., 2022). These transplanted macrophages enhance debris clearance, secrete growth factors, and support nerve regeneration. These strategies highlight the potential of macrophage modulation as a therapeutic approach for peripheral nerve repair by optimizing macrophage phenotype dynamics to facilitate regeneration.

The induction of M2 macrophages has also been explored as a therapeutic strategy in animal models of autoimmune neuropathy and traumatic nerve injury. Dimethyl fumarate (DMF) exerts neuroprotective effects by shifting the immune response from Th1 to Th2, increasing IL-10 and IL-4 levels, inhibiting NF-κB activity, and reducing inflammatory lymphocyte activity. In EAN, DMF upregulates heme oxygenase-1 (HO-1) and Nrf2, promoting M2 polarization (Han et al., 2016b). Compound A, a plant-derived glucocorticoid receptor ligand, enhances recovery in EAN by increasing anti-inflammatory macrophages (Zhang et al., 2009). Another pharmacological agent, everolimus (RAD001), an mTOR inhibitor, alleviates EAN symptoms by inducing an Akt-mediated shift toward the M2 phenotype while increasing IL-4 and TGF-β production, reinforcing the anti-inflammatory response (Han et al., 2016a). Additionally, Bowman-Birk inhibitor concentrate (BBIC), a soybean-derived protease inhibitor, reduces autoimmune responses and disease severity in EAN by promoting M2 macrophage polarization, upregulating IL-10 and other anti-inflammatory cytokines, and suppressing proinflammatory mediators (Jin et al., 2016). In traumatic nerve injury models, mesenchymal stem cell-derived exosomes containing miRNAs and growth factors have been shown to shift macrophages toward an M2-like phenotype, reducing fibrosis and improving nerve repair (Li et al., 2022). Metformin, a widely used antidiabetic agent, has also been demonstrated to enhance functional recovery, axon regeneration, and remyelination by activating the AMPK/PGC-1α/PPAR-γ signaling axis, promoting M2 polarization in a peripheral nerve injury model (Zhou et al., 2023).

While the promotion of M2 macrophages is beneficial for resolving inflammation and aiding tissue repair, it is important to recognize that M1 macrophages play a vital role in the early stages of injury by clearing debris. Therefore, a carefully timed transition from M1 to M2 polarization is necessary to ensure optimal healing without compromising debris clearance. Further research is required to determine the ideal timing and dosage of these interventions to maximize their therapeutic potential.

Human pathological studies, as well as data derived from various animal models, strongly indicate that macrophage lineage cells serve as the ultimate effectors in mediating nerve injury in autoimmune neuropathies (Asbury et al., 1969; Hartung et al., 1988; Heininger et al., 1988; Hafer-Macko et al., 1996). One of the key regulatory factors in macrophage biology is the colony-stimulating factor 1 receptor (CSF1R), which is essential for the proliferation, differentiation, survival, and functional activity of monocytes and fully differentiated macrophages. CSF1R activation by its primary ligand, colony-stimulating factor 1 (CSF1), or other related ligands, facilitates various macrophage-mediated processes, including their recruitment and transmigration into affected tissues during inflammatory responses (Pixley and Stanley, 2004; Pixley, 2012; Stanley and Chitu, 2014). Given its pivotal role, CSF1R signaling is a critical determinant in shaping macrophage responses in both homeostatic and pathological conditions. In our prior studies, we showed that CSF1-deficient osteopetrotic mice, that are deficient in monocytes and tissue macrophages, were resistant to anti-ganglioside antibodies-mediated nerve injury in two separate animal models of inflammatory neuropathy (Zhang et al., 2014; He et al., 2015). By blocking CSF1R, we can potentially reduce the infiltration and accumulation of endoneurial macrophages, thereby mitigating the inflammatory burden within the peripheral nerve and limiting the extent of nerve injury. Such an approach could have profound implications for developing novel treatment strategies aimed at suppressing macrophage-driven nerve inflammation and preserving neuronal function in peripheral neuropathies.

Other immunomodulatory strategies beyond macrophage modulation target regulatory T cells (Tregs), B cells, complement activation, and autoantibodies. Tregs support nerve repair by secreting anti-inflammatory cytokines and interacting with macrophages. Study has shown that CD4-targeted nanoparticles loaded with IL-2 and TGF-β effectively promote Treg expansion *in vivo* (McHugh et al., 2015). Notably, Tregs expansion through leflunomide-based therapies or adoptive Treg transfer reduces excessive inflammation in animal models of autoimmune neurological disorders like Multiple Sclerosis and GBS (Korn et al., 2001; Ochoa-Reparaz et al., 2016). Therapies targeting autoantibody-producing B cells, such as anti-CD20 (Rituximab, a murine-human chimeric monoclonal antibody against CD20), can be beneficial in autoantibody mediated autoimmune polyneuropathies where excessive inflammation hinders repair (Querol et al., 2015; Jiao et al., 2020). Complement inhibitors (e.g., Eculizumab, a humanized monoclonal antibody against the complement component C5) can block complement-mediated nerve injury, particularly in conditions like multifocal motor neuropathy (Fitzpatrick et al., 2011). Efgartigimod, an inhibitor of neonatal Fc receptors, enhances IgG clearance and has demonstrated clinical efficacy in CIDP, with a phase II study showing symptom improvement in 67% of patients (Allen et al., 2024), many of whom had been withdrawn from intravenous immunoglobulin therapy.

Incorporating immunomodulatory agents into nerve grafts presents a promising strategy to enhance peripheral nerve repair by modulating the inflammatory response and promoting a regenerative microenvironment. In cases where nerve trauma results in a gap exceeding 5 mm or severe nerve damage (Sunderland grade 3–5), surgical interventions such as nerve grafting are necessary (Yeoh et al., 2022), with autologous nerve grafting being the gold standard for defects greater than 3 cm (Faroni et al., 2015). Studies have demonstrated that integrating immunomodulatory agents, such as FK506, into nerve grafts significantly improves functional recovery compared to conventional grafting alone (Doolabh and Mackinnon, 1999; Zuo et al., 2021). Additionally, mesenchymal stem cells (MSCs) have emerged as a novel therapeutic approach, leveraging their ability to secrete immunoregulatory factors and modulate both innate and adaptive immune responses (Li et al., 2022). MSCs influence immune cells, including macrophages, natural killer cells, and T and B lymphocytes, through paracrine signaling and direct cell-to-cell interactions, facilitating the transition from a pro-inflammatory to a pro-regenerative environment. The secretion of bioactive molecules such as cytokines, chemokines, and growth factors plays a crucial role in their therapeutic potential, often exceeding their differentiation capability (Siniscalco et al., 2010). While the immunomodulatory effects of MSCs in peripheral nerve injury remain incompletely understood, accumulating evidence suggests that these mechanisms contribute significantly to nerve regeneration, highlighting the potential of immunomodulatory strategies in optimizing nerve graft outcomes (Lavorato et al., 2021).

**FIGURE 3:**
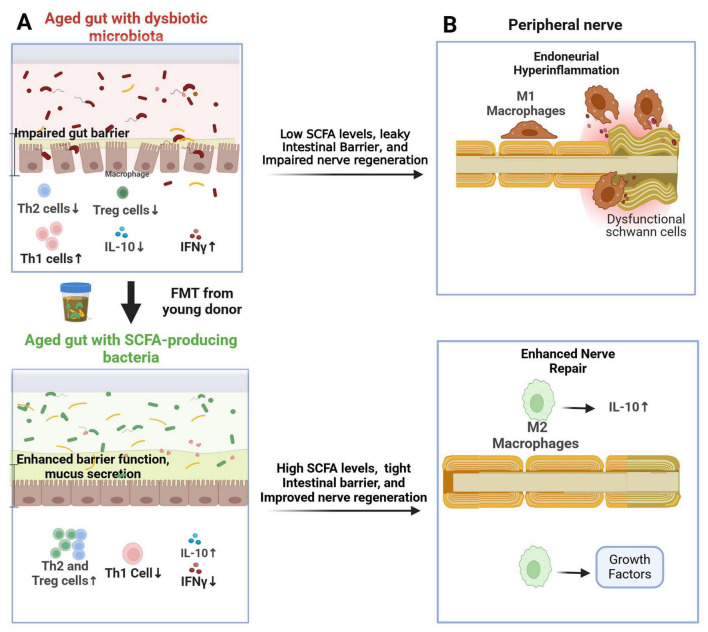
Proposed role of the gut microbiome in nerve repair following peripheral nerve injury. **(A)** Rejuvenating fecal microbiota transplantation (FMT) from young donors restores a youthful gut microbiome in aged recipients, leading to increased fecal short-chain fatty acid (SCFA) levels. This promotes the activation of Th2 and Treg cells in the gut mucosa and spleen, resulting in elevated IL-10 levels; **(B)** In aged mice, the youthful gut microbiome enhances the response of IL-10-producing M2 macrophages in the endoneurium, mitigating aging-related endoneurial hyperinflammation (“inflammaging”) and facilitating nerve repair.

The gut microbiota and its metabolites, particularly short-chain fatty acids (SCFAs), play a crucial role in regulating host immunity (Belkaid and Hand, 2014). Altered gut microbiota, observed in aging individuals and certain disease states, can contribute to a pro-inflammatory environment and impair nerve regeneration (Davari et al., 2013; Yang et al., 2015; Singh et al., 2016; Lee et al., 2020; Svacina et al., 2024a). Animal studies have shown that gut microbiota modulation, via antibiotics or probiotics, ameliorates EAN by reducing endoneurial inflammation (Meng et al., 2023; Sprenger-Svacina et al., 2024a). Additionally, rejuvenating fecal microbiota transplantation (FMT) in aged mice undergoing sciatic nerve crush injury induced an anti-inflammatory Th2 response, leading to significantly enhanced functional, electrophysiological, and histological nerve recovery (Figure 3; Svacina et al., 2024a). This improvement correlated with increased endoneurial M2 macrophages three weeks post-injury. Beyond established immunomodulatory drugs for immune neuropathies (e.g., intravenous immunoglobulins), gut microbiota interventions may represent a novel strategy for enhancing peripheral nerve repair (Rodenhouse et al., 2022; Svacina and Lehmann, 2022; Svacina et al., 2024a).

Tissue-resident macrophages and BDMs have distinct yet complementary roles in peripheral nerve injury and repair (Sprenger-Svacina et al., 2024b). TRMs, located within the endoneurium, contribute to immune surveillance and homeostasis, providing an early response to injury. In contrast, BDMs are recruited post-injury, playing a dominant role in debris clearance, inflammation resolution, and regeneration support. A balanced interaction between these macrophage populations is crucial for optimal nerve repair, and therapeutic modulation of their activity presents a promising avenue for improving recovery. Recent findings from our group suggest that macrophage origin significantly influences endoneurial immune responses and nerve repair (Sprenger-Svacina et al., 2024b). Specifically, TRMs appear to play a more prominent role in sustaining chronic endoneurial inflammation in SAPP compared to BDMs. This insight highlights the potential for targeting TRMs in macrophage-based therapies aimed at mitigating chronic inflammation and improving nerve regeneration.

Immunomodulatory strategies offer promising avenues for improving nerve repair by balancing inflammation and regeneration. However, peripheral nerve repair is a multifaceted process requiring the integration of immune modulation, neurotrophic support, and structural guidance. Future research should focus on combinatorial approaches to optimize outcomes in both traumatic and autoimmune nerve injuries.

## Conclusion

Peripheral nerve injuries trigger a highly dynamic and coordinated series of events that govern tissue degeneration and subsequent repair. Understanding these intricate mechanisms is essential for developing targeted therapeutic strategies. This review highlights the critical role of immune responses, particularly macrophages, in orchestrating nerve repair and explores emerging therapeutic avenues for modulating immune function to optimize regeneration. PNIs can arise from diverse etiologies (Kieseier et al., 2018; Sommer et al., 2018). Each of these conditions leads to nerve damage through distinct mechanisms that influence both the injury pattern and the repair process. A fundamental distinction exists between primary axonal injuries, characterized by direct axonal transection or metabolic degeneration, and demyelinating injuries, where myelin sheaths are selectively targeted. This diversity underscores the complexity of peripheral nerve pathologies and highlights the need for tailored treatment approaches that account for injury-specific cellular responses.

The pathophysiological response to PNI involves a cascade of molecular and cellular events, including axonal degeneration, myelin breakdown, immune activation, and subsequent tissue remodeling (Gaudet et al., 2011). Wallerian degeneration plays a pivotal role in clearing myelin and axonal debris, while Schwann cells exhibit remarkable plasticity, dedifferentiating into repair-supportive phenotypes that promote axon regeneration and remyelination. Among immune cells, macrophages serve as central regulators of this process, participating in both destructive and reparative phases of nerve injury. Their plasticity enables them to transition between a pro-inflammatory state, which is crucial for debris clearance and pathogen defense, and a pro-regenerative state, which fosters tissue remodeling and axonal regrowth.

However, disruptions in the immune response can severely impede nerve repair. An insufficient macrophage response may lead to incomplete clearance of inhibitory debris, delaying axonal regeneration, whereas an exaggerated or persistent inflammatory response can create a neurotoxic microenvironment. This imbalance is particularly evident in conditions such as CIDP and age-related “inflammaging,” where prolonged immune activation contributes to nerve dysfunction and impaired recovery (Buttner et al., 2018). Thus, fine-tuning immune responses, particularly macrophage activation states, represents a promising therapeutic strategy for improving nerve repair outcomes.

The role of macrophage subsets in tissue injury and repair is complex and context-dependent, with blood-derived and tissue-resident macrophages exhibiting distinct yet sometimes overlapping functions (Wynn and Vannella, 2016). BDMs are well-recognized for their role in debris clearance and tissue repair following injury, whereas the function of TRMs remains controversial. In the context of autoimmune neuropathies, TRMs have been implicated in sustaining chronic inflammation, potentially exacerbating tissue damage and contributing to disease progression (Sprenger-Svacina et al., 2024b). However, studies in cardiac tissue suggest a contrasting role, where embryonically derived tissue-resident macrophages facilitate the resolution of inflammation and promote repair (Lavine et al., 2014). This discrepancy may arise from differences in tissue-specific macrophage programming, micro environmental cues, or the nature of the inflammatory triggers. In the heart, homeostatic conditions may favor an anti-inflammatory phenotype, while in neuropathies, persistent autoimmune activation could drive tissue-resident macrophages toward a pathogenic role. Understanding these distinctions is crucial for developing targeted immunomodulatory therapies that can selectively modulate macrophage functions to control inflammation while preserving regenerative potential.

Several promising therapeutic strategies are being explored to enhance nerve regeneration by modulating immune responses: (1). Macrophage Polarization Modulation: Macrophage modulation represents a promising avenue for peripheral nerve repair. Therapeutic strategies focusing on suppressing inflammatory M1 activity, enhancing M2 polarization, and leveraging macrophage-based cell therapies continue to advance. Innovations in immunomodulatory treatments, including cytokine delivery, lipid mediators, and pharmacological interventions, are refining approaches for optimizing macrophage phenotype dynamics to foster nerve regeneration. Facilitating a timely transition from M1 to M2 macrophages through targeted immunotherapies may further enhance tissue repair and improve clinical outcomes. (2). Integrating Immunotherapy with Surgical Interventions: The combination of immunomodulatory strategies with surgical approaches, such as nerve grafts or conduits, holds significant potential for optimizing nerve repair. Immunomodulation plays a crucial role in this process by balancing pro- and anti-inflammatory responses to create a regenerative microenvironment. For instance, calcineurin inhibitors have been employed to improve nerve graft acceptance and enhance functional recovery by suppressing excessive immune activation (Doolabh and Mackinnon, 1999; Zuo et al., 2021). Similarly, MSC-based therapies, when used in conjunction with nerve guide conduits, contribute to nerve regeneration through immunomodulation (Dong et al., 2019; Li et al., 2022). MSCs regulate immune responses, secrete extracellular matrix proteins and trophic factors, and support remyelination of regenerating axons, ultimately fostering a more favorable environment for nerve repair (Praveen Kumar, et al., 2019; Lavorato et al., 2021; Li et al., 2022). (3). Gut Microbiota Modulation: Emerging evidence suggests that gut microbiota influence systemic immune responses and may impact peripheral nerve repair (Rodenhouse et al., 2022; Svacina et al., 2024a). Probiotic supplementation and FMT have been investigated as potential strategies to modulate the immune milieu, creating an anti-inflammatory environment conducive to nerve regeneration. (4). Targeting Specific Immune Pathways: Blocking key pro-inflammatory pathways has demonstrated therapeutic potential in experimental models. For instance, inhibiting Fc-gamma receptors on macrophages (Zhang et al., 2014; Zhang and Sheikh, 2017; Zhang et al., 2019) or disrupting the CSF1/CSF1R axis (Zhang et al., 2014; He et al., 2015) has shown promise in reducing endoneurial macrophage burden and promoting nerve repair. These approaches may hold clinical relevance for treating immune-mediated neuropathies.

Peripheral nerve repair is a complex and tightly regulated process influenced by immune responses, Schwann cell activity, and the endoneurial microenvironment. The immune microenvironment within injured nerves plays a decisive role in determining repair outcomes, necessitating a delicate balance between pro-inflammatory and regenerative processes. While macrophages are central to these immune dynamics, disruptions in their polarization and activation states can hinder recovery, contributing to chronic neuropathies or suboptimal healing. Therapeutic strategies aimed at fine-tuning immune responses, particularly macrophage modulation, represent a promising frontier in nerve repair research. Additionally, novel approaches such as gut microbiota interventions and biomaterial-based immunomodulation may further enhance regenerative capacity. By integrating these strategies with surgical interventions and emerging immunotherapies, it may be possible to improve functional outcomes for patients with peripheral nerve injuries, paving the way for more effective and personalized treatment paradigms.
